# Survival of indirect pulp capping in deeply carious primary molars under local versus general anesthesia: a retrospective cohort study using propensity score matching

**DOI:** 10.1186/s12903-026-08019-w

**Published:** 2026-03-11

**Authors:** Qiyin Sun, Xinling Liang, Mianxiang Li

**Affiliations:** Guangzhou Women and Children’s Medical Center, Guangzhou Medical University, Guangzhou, China

**Keywords:** Indirect pulp capping, Deep caries, Primary molar, Propensity score matching

## Abstract

**Objectives:**

This study aimed to evaluate the long-term efficacy of indirect pulp capping performed under general anesthesia or local anesthesia in primary molars with deep caries. It also aimed to investigate factors influencing the success rate.

**Methods:**

The medical records of children with deeply carious primary molar, who received indirect pulp capping under general anesthesia or local anesthesia from 2022, were screened and collected. Propensity score matching method was used to match the general anesthesia and local anesthesia groups 1:1 based on covariates. Kaplan-Meier survival analysis was conducted and Cox proportional hazards model was applied to analyze the outcomes.

**Results:**

Propensity score matching resulted in 120 pairs of matched teeth from 666 teeth meeting inclusion criteria. Subsequently, a total of 14 failures (5.83%) were observed. Kaplan–Meier survival curves and the log-rank test revealed no observable difference in the success rates of indirect pulp capping performed under the two anesthesia methods (adjusted HR: 1.85, 95% CI: 0.62–5.51). In the analysis of the cohort of all teeth, the Cox proportional hazards model showed observable associations between indirect pulp capping and tooth type (HR: 0.34, 95% CI: 0.15–0.77), and restoration technique (HR: 0.48, 95% CI: 0.24–0.97). Age, gender, arch position, decayed tooth surface and anesthesia method had no observable effect on indirect pulp capping success.

**Conclusion:**

In this study, the overall survival rate of indirect pulp capping did not decrease observably with time, irrespective of general anesthesia or local anesthesia. Additionally, tooth type and restorative technique are independent prognostic factors for the survival.

**Supplementary Information:**

The online version contains supplementary material available at 10.1186/s12903-026-08019-w.

## Introduction

Primary dental caries is a major public health concern with a very high incidence rate of 60%–90% in the world. It can easily trigger localized dental infection along with systemic infection if not treated promptly [1]. Dental caries in primary teeth not only causes pain and infection, affecting nutritional intake and sleep, but also damages the developing permanent tooth buds. Additionally, loss of chewing stimulus and premature primary tooth loss may lead to malocclusion [2]. Primary molars, as the prominent teeth for mastication, space maintenance, and permanent tooth bud eruption guidance, have higher caries incidence and more serious negative effects. Pulp-preserving treatment is the treatment of preference for deep caries in primary molars with healthy pulp or reversibly inflamed pulp. It maximizes the preservation of vitality of the primary tooth until spontaneous exfoliation and prevents damage to the permanent tooth bud [3].

Pulp-preserving treatments include direct pulp capping, indirect pulp capping (IPC), and pulpotomy. IPC avoids direct injury to the pulp, allowing it to spontaneously form reparative dentin. Furthermore, the procedure is less technically demanding, more minimally invasive, and causes less iatrogenic damage. For pediatric patients, it also results in shorter treatment duration and improved acceptability [3, 4]. Stratigaki et al. [5] enrolled nine systematic reviews on the treatment of deep caries in primary teeth and found that IPC demonstrated the highest success rates at 24 months (94%) compared to pulpotomy (82.6%). According to the American Academy of Pediatric Dentistry (AAPD) guidelines [6], among all vital pulp therapies, IPC has the highest overall success rate at 24 months (97%). Therefore, for deep caries in primary molars with healthy pulp or only reversible inflammation, the physiologically sound and cost-effective treatment approach of selective debridement combined with IPC is recommended as the preferred option.

The diagnosis of pulp status in primary teeth is a challenging task due to the immature development of pulp nerves and the unreliability of children’s responses, which is particularly difficult in young children [7]. In addition, the efficacy of IPC has been stated to be correlated with tooth position, arch position, and restorative techniques [8, 9]. General anesthesia (GA) treatment provides a superb operating field, effectively guarding against patient and saliva interference with the dentist. The technology can address all the oral issues in a single visit and is a very favourable option when patient compliance issues compromise treatment outcomes [10]. Previous studies have examined the association between IPC success rates and factors such as age, preoperative sensitivity, tooth type, number of surfaces with caries, color of cavity floor and whether the pulp is exposed [11]. However, few studies have investigated the IPC survival rate in deep primary molar caries and its risk factors, especially from the perspective of long-term observation and large sample size. Moreover, clinical studies comparing the IPC survival analyses of deep caries in molars under local anesthesia (LA) and GA are limited.

Accordingly, the goal of this research is to comprehensively compare the long-term efficacy of IPC for primary molar deep caries treatment under GA and LA in a retrospective cohort study and determine influencing factors for the rate of treatment success. The outcome will provide practitioners and parents with more reliable evidence-based suggestions for selecting the most appropriate treatment methods and assessing tooth prognosis. This will allow for customized, precision-guided therapy of deep caries in primary molars, ultimately leading to enhanced pediatric oral health outcomes.

## Method

The Guangzhou Women and Children’s Medical Center’s Medical Ethics Committee approved this study (SFEKL [2025] NO.557B00), which was carried out in compliance with the principles of the Declaration of Helsinki. As the data for this retrospective study, extracted from previous medical records, were recorded in a way that did not identify the subjects, consent was not required. Parental informed consent was obtained before dental visit and operation, and parents were explicitly informed that information in the medical records may be collected for academic research purposes in the future.

This retrospective cohort study was conducted and reported in accordance with the Strengthening the Reporting of Observational Studies in Epidemiology (STROBE) guideline. A completed STROBE checklist is provided as Supplementary Material 1.

### Subject selection

Eligible dental samples were selected according to predefined inclusion criteria. This study included cases of IPC performed under GA or LA in Department of Stomatology, Guangzhou Women and Children’s Medical Center, Guangzhou Medical University from January to December 2022, all involving children diagnosed with deep caries in their deciduous molars. To fully evaluate the long-term efficacy of IPC, we collected follow-up data until June 2025.

The inclusion criteria for the study are as follows:

1. Healthy children aged 3–8 years.
2. Ability to communicate with dentists.
3. At least one restorable primary molar is diagnosed with deep caries, and there is no spontaneous unprovoked toothache.
4. Deep caries was diagnosed according to the criteria of the European Society of Endodontology (ESE): Caries reaches the inner quarter of dentine, with a detectable hard-dentine zone between the caries and the pulp on interproximal or occlusal radiographs. There is a risk of pulp exposure during treatment [12].
5. The teeth should show no signs of internal resorption, thickening of the periodontal spaces, or radiolucency surrounding the tooth apex or the furcation in the radiograph.
6. The treated tooth should have a preoperative radiograph and at least one radiographic assessment after the treatment every three to six months.

Exclusion criteria for the study are as follows:

1. Patients with systemic disease or mental health problems.
2. Clinical symptoms of irreversible pulpitis, such as a history of spontaneous, persistent, and/or nocturnal pain.
3. Tenderness to percussion or abnormal mobility.
4. Fistula, or gingival swelling on clinical examination.
5. Interradicular or periapical radiolucencies and root resorption (pathological internal/external or physiological).
6. Extensive caries requiring extraction.

### Treatment procedure

The choice of anesthesia modality (LA vs. GA) was determined through shared clinical decision-making between pediatric dentists and parents/guardians. Local anesthesia was typically employed for cooperative children (Frankl Scale 3–4) with isolated lesions amenable to single-visit treatment. General anesthesia was indicated for: (1) pre-cooperative or uncooperative children (Frankl Scale 1–2); (2) very young patients (< 4 years) requiring complex procedures; (3) children with special health care needs; (4) extensive caries requiring multiple quadrant treatment exceeding the tolerance limits for chairside care; or (5) previous failed attempts under LA. The final decision incorporated parental preferences after informed consent regarding anesthesia risks and benefits.

GA was arranged in the day surgery center and conducted in accordance with an established protocol [13]. Dental treatment under LA is carried out in the department for outpatients.

First, all the surface carious lesions and undermined enamel were excavated with a high-speed handpiece. The lesion on the lateral walls and cavosurface margins was removed completely with a low-speed bur. The lesions on the pulp wall were selectively removed, and the operator carefully avoided exposing the pulp. The prepared cavity was slightly rinsed and dried with air/water spray. Then pulp capping material (Calcimol LC, VOCO, Germany) was applied to the pulp wall nearest to the pulp. Choice of restoration method depended on the size of the defective area. The preformed metal crown (Kids Crown, ShinhungCo., Ltd, Korea) with GIC was routinely selected for molars where more than two surfaces were affected, or where one or two surface carious lesions were extensive. If the cavity was limited or single-sided, resin composite material (Gradia Direct, GC, Japan) could be used directly for repair.

Follow-up checks are required every 3–6 months after the operation, including clinical and radiological examinations. If the patient is visiting the dentist due to discomfort, periapical radiographs should be taken according to their complaints and symptoms.

### Data collection

The study systematically collected data including patients’ social-biological characteristics, examination records, and follow-up details. Sociobiological characteristics included patient name, gender, age, and treatment date. Examination details covered tooth type (1st primary molar or 2nd primary molar), arch position (maxillary or mandibular), whether caries involves the mesial surface, and radiographic findings. Follow-up data included follow-up dates, primary complaints, crown restoration condition, clinical manifestations, and radiographic findings such as root bifurcation or periapical shadows, root resorption. Behavioral assessments using validated scales (e.g., Frankl Behavior Rating Scale) were not systematically documented in available records, precluding inclusion in propensity score models. GA indication was inferred from clinical notes referencing ‘uncooperative behavior,’ ‘failed previous attempts,’ or ‘multiple quadrants requiring treatment.’

The outcome of this study was evaluated both clinically and radiographically. The two researchers independently evaluated the clinical and radiographic findings. If the two researchers disagreed, Professor Sun was asked to resolve the issue. A pilot test was conducted on 20 randomly selected images. The Cohen’s kappa values for the internal agreement of the two researchers were 0.84 and 0.87, and the external agreement between their evaluations was 0.78.

Criteria for the outcome for the study are as follows:

1. Clinical criteria for success.

- No spontaneous discomfort or pain during percussion was experienced.
- The adjacent soft tissues were healthy, with no signs of swelling, abscess, or sinus tract.
- No abnormal mobility was detected.
- Normal physiological functions were observed.

2. Success criteria for radiographic examination.

- No pathological alterations in the periodontium, root furcation, or periapical regions were present.
- No indications of pathological lesions in the succeeding permanent teeth were found.
- No signs of pathological root resorption were evident.
- The treatment will be deemed unsuccessful if any of the above mentioned criteria (whether clinical or radiographic) are not fulfilled [14].

### Statistical analysis

This study employed two distinct analytical approaches based on different research objectives:

1. Primary analysis: To compare the efficacy of IPC performed under LA versus GA, we used propensity scores matching (PSM) to balance baseline covariates between groups, allowing for a robust causal comparison while minimizing confounding bias. We performed 1:1 nearest neighbor PSM with a caliper of 0.02 to balance baseline characteristics between the LA and GA groups. The propensity scores were estimated using a multivariate logistic regression model incorporating the following covariates: gender, age, arch position, tooth type, and restoration technique.These variables were selected based on: (a) clinical plausibility as potential confounders affecting anesthesia choice, dental characteristics and surgical outcomes; (b) existing literature demonstrating associations with postoperative complications [14, 15]; (c) variables accurately retrievable from clinical record; and (d) data completeness (> 95% available records).
2. Secondary analysis: To identify factors associated with IPC success, we performed exploratory Cox regression using the entire cohort of all teeth meeting inclusion criteria. This approach maximized statistical power and preserved the full spectrum of clinical variability necessary for predictor identification. We employed the change-in-estimate method to select confounders. Starting from a full model with all potential confounders, we sequentially removed each variable and retained those causing ≥ 10% change in the exposure effect estimate (Table S1). Variables of known clinical importance were forced into the final model.
3. Exploratory supplementary analysis: We stratified by anesthesia method (LA vs. GA) to explore whether predictors behave differently within each anesthesia subgroup. This is an exploratory supplementary analysis to assess effect modification.

PASS 15 software was utilized to calculate the required sample size. Using *t* tests with the statistical test “Difference between two dependent means (matched pairs)’’, an effect size of 0.30, an α = 0.05, and a power of 0.90, the total required sample size was 172, necessitating a minimum of 86 samples per group. This calculation is appropriate for determining sample sizes for groups matched through PSM.

The Shapiro–Wilk test was used to evaluate whether the quantitative measurements followed a normal distribution. Normally distributed continuous variables were presented as means (with standard deviation), while categorical variables were reported as counts with percentages. The weighted Student’s *t*-test and chi-square test were used to compare different groups. The aforementioned statistical analyses were conducted with SPSS 27.0, with a 2-tailed alpha level of 0.05 specified for all analyses.

Using R statistical computing software (version 4.4.1), Kaplan–Meier survival analysis was performed to estimate survival times, which were depicted through cumulative survival curves. Log-rank test was used to assess the treatment success across groups. The Cox proportional hazards model was used to analyze factors influencing success rates, in which *P* < 0.05 was regarded as indicating an observable association.

## Result

**Fig. 1:**
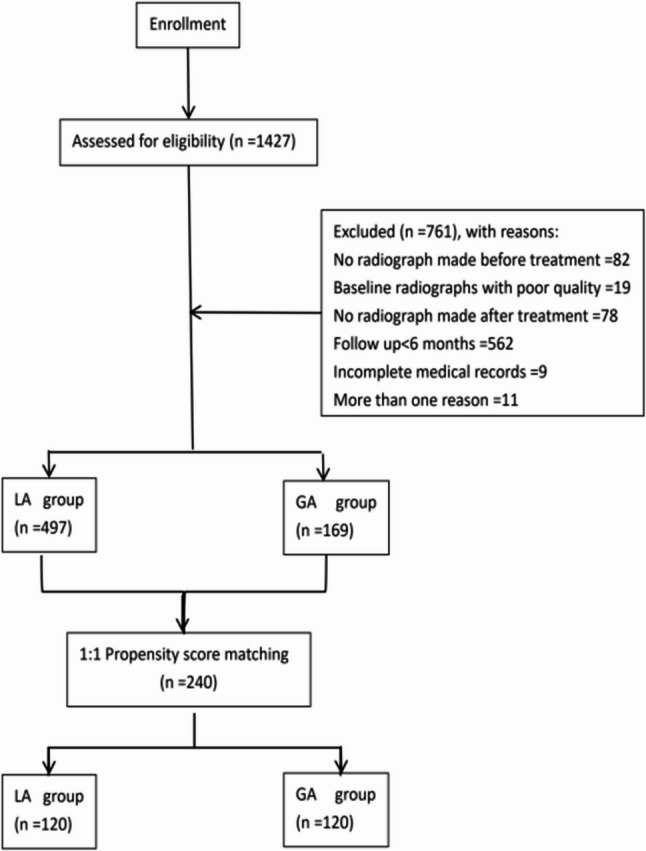
Flowchart of the retrospective study

In 2022, a total of 483 patients with 1,427 primary molars underwent IPC. Based on the inclusion and exclusion criteria, some patients were excluded because of missing follow-up data, incomplete medical records, or lack of radiographic evaluation. Ultimately, the study included 286 patients with 666 primary molars. Among them, 497 teeth were in the LA group and 169 teeth were in the GA group. Follow-up duration was 28.84 ± 5.48 months. Considering the baseline differences and notable variations in case numbers between groups, we used the PSM method to improve comparability and reduce selection bias. By performing 1:1 matching with gender, age, tooth type, arch position, anesthesia method, and restoration as covariates, we obtained 240 matched tooth pairs. This accurate calculation meets the sample size requirements. A flowchart is illustrated in Fig. 1.

1. Primary outcomes: The baseline characteristics of teeth before and after PSM are summarized in Table 1. After PSM, all standardized mean differences(SMD) were < 0.20, and *P* > 0.05 for all included covariates. No notable standardized differences were observed for any baseline covariates post-matching. The comparison of propensity score distribution before and after matching is shown in Fig.S1.

**Table 1:** Baseline characteristics analysis according to anesthesia method before and after PSM

Variable	Before PSM				After PSM			
	LA	GA	SMD	*P*-value	LA	GA	SMD	*P*-value
	*n* (%)	*n* (%)			*n* (%)	*n* (%)		
Age (Years), mean (SD)	5.10 (1.27)	4.32 (1.11)	0.67	0.00*	4.97 (1.17)	4.73 (0.99)	0.09	0.55
Gender, n (%)			0.29	0.00*			0.14	0.32
Male	243 (36.49)	107 (16.07)			68 (28.33)	88 (36.67)		
Female	254 (38.14)	62 (9.31)			52 (21.67)	32 (13.33)		
Tooth type, n (%)			0.05	0.59			0.12	0.90
1^st^ primary molar	249 (37.39)	89 (13.36)			57 (23.75)	59 (24.58)		
2^nd^ primary molar	248 (37.24)	80 (12.01)			63 (26.25)	61 (25.42)		
Arch position, n (%)			0.09	0.29			0.08	0.57
Maxillary arch	255 (38.29)	95 (14.26)			62 (25.83)	71 (29.58)		
Mandibular arch	242 (36.34)	74 (11.11)			58 (24.17)	49 (20.42)		
Restoration, n (%)			1.97	0.00*			0.00	1.00
Resin composite	387 (58.11)	14 (2.10)			21 (8.75)	14 (5.83)		
Stainless steel crown	110 (16.52)	155 (23.27)			99 (41.25)	106 (44.17)		
Total, n (%)	497 (74.62)	169 (25.38)			120 (50.00)	120 (50.00)		

*PSM* propensity score matching, *LA* local anesthesia, *GA* general anesthesia, *SMD* standardized mean differences, *SD* standard deviation

*significance value

14 failures (5.83%) were observed in teeth during the follow-up period. The average time to failure was 19.21 ± 6.82 months. The univariate analysis (Table 2) showed an observable association between failures and arch position (*P* = 0.03) and restorative technique (*P* = 0.04).

**Table 2:** Potential factors affecting the success rate of IPC (*N* = 240)

Variable	Failure	Success	*P*-value
	*n* (%)	*n* (%)	
Age (Years), mean (SD)	4.86 (0.95)	4.85 (1.09)	0.98
Gender, n (%)			0.78
Male	10 (4.17)	146 (60.83)	
Female	4 (1.67)	80 (33.33)	
Tooth type, n (%)			0.28
1^st^ primary molar	9 (3.75)	107 (44.58)	
2^nd^ primary molar	5 (2.08)	119 (49.58)	
Arch position, n (%)			0.03*
Maxillary arch	12 (5.00)	121 (50.42)	
Mandibular arch	2 (0.83)	105 (43.75)	
whether caries involves the mesial surface, n (%)			0.65
No	2 (0.83)	24 (10.00)	
Yes	12 (5.00)	202 (84.17)	
Anesthesia method, n (%)			0.41
LA	5 (2.08)	115 (47.92)	
GA	9 (3.75)	111 (46.25)	
Restoration, n (%)			0.04*
Resin composite	5 (2.08)	30 (12.50)	
Stainless steel crown	9 (3.75)	196 (81.67)	
Total, n(%)	14 (5.83)	226 (94.17)	

*SD *standard deviation, *LA *local anesthesia, *GA* general anesthesia

*significance value

**Fig. 2:**
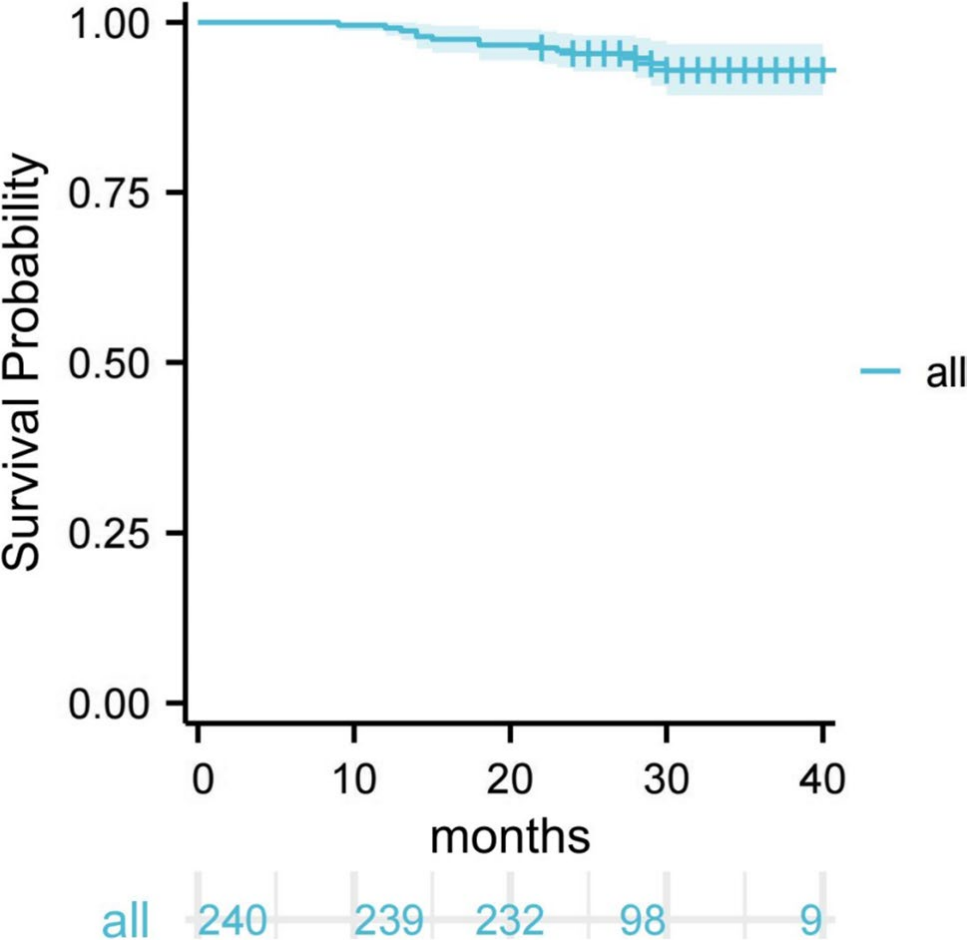
Survival analysis of all teeth

**Fig. 3:**
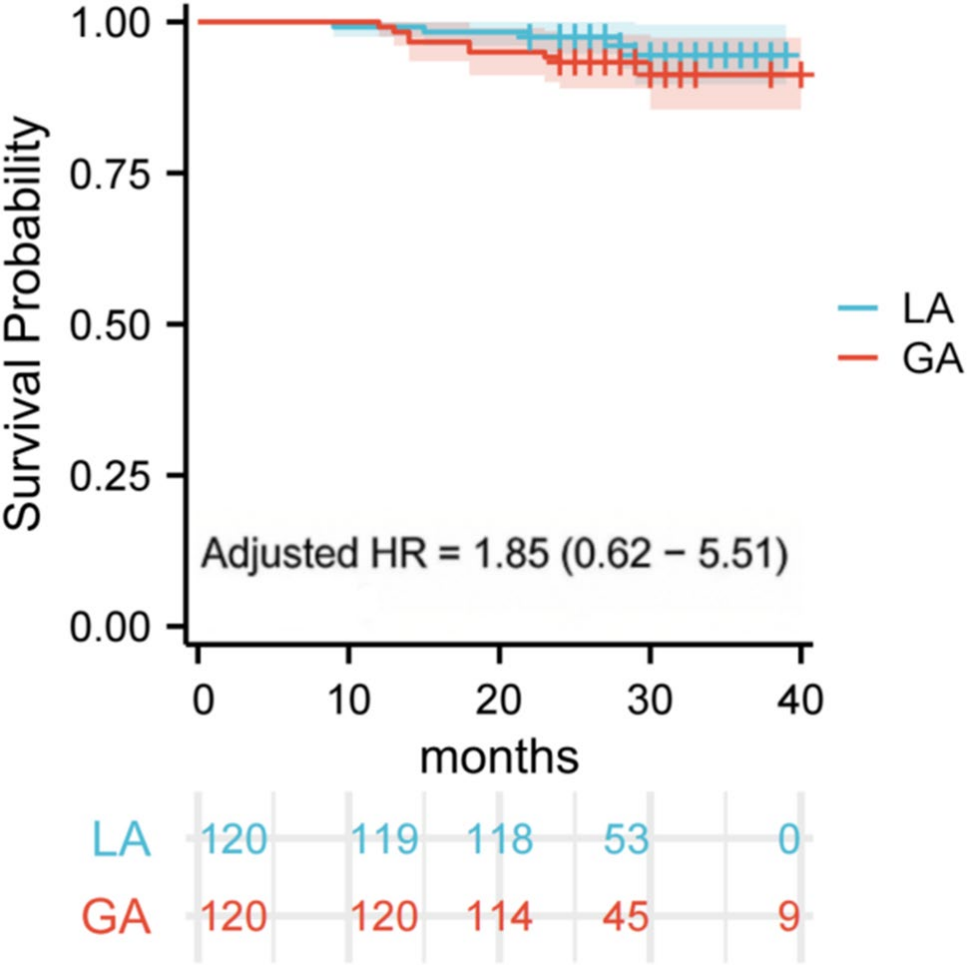
Survival analysis of the teeth under two anesthesia methods

The Kaplan-Meier survival analysis of IPC long-term efficacy in all teeth after PSM is presented in Fig. 2. The survival rates for 6 months, 12 months, 18 months, 24 months, 30 months, 36 months, and 40 months were 100.00%, 99.20%, 96.70%, 95.40%, 93.00%, 93.00% and 93.00%, respectively (Fig. 2). Figure 3 shows Kaplan–Meier survival curves comparing IPC survival between the LA and GA groups. The HR, CI were derived from the multivariable Cox proportional hazards model, adjusted for age, gender, tooth type, arch position, whether caries involves the mesial surface and restoration. The log-rank test results revealed no observable difference between LA and GA (adjusted HR: 1.85, 95% CI: 0.62–5.51). This indicates that different anesthetic methods do not affect the long-term efficacy of IPC.

2. Secondary outcomes: In the analysis of the cohort of 666 teeth meeting inclusion criteria, the Cox proportional hazards model revealed an association. Specifically, there were associations between the survival time of teeth treated with IPC and tooth type (HR: 0.34, 95% CI: 0.15–0.77), as well as between the survival time and restoration technique (HR: 0.48, 95% CI: 0.24–0.97) (Table 3). By contrast, gender, age, arch position, anesthesia method, and mesial surface caries showed no detectable effects on surgical success rate.

**Table 3:** Cox proportional hazards model analysis of factors influencing success rate (*N* = 666)

	Unadjusted HR^1^ (95% CI)	Adjusted HR^2^ (95% CI)
Age	0.95 (0.71–1.27)	
Gender		
Male	Ref.	
Female	0.71 (0.34–1.46)	
Tooth type		
1^st^ primary molar	Ref.	Ref.
2^nd^ primary molar	0.34 (0.15–0.76)	0.34 (0.15–0.77)
Arch position		
Maxillary arch	Ref.	
Mandibular arch	0.92 (0.45–1.87)	
whether caries involves the mesial surface		
No	Ref.	
Yes	1.96(0.47–8.20)	
Anesthesia method		
LA	Ref.	
GA	0.70 (0.29–1.70)	
Restoration		
Resin composite	Ref.	Ref.
Stainless steel crown	0.66 (0.43–1.00)	0.48 (0.24–0.97)

*HR* hazard ratio, *CI* confidence interval, *Ref* reference, *LA *local anesthesia, *GA* general anesthesia

^1^Values are regression coefficients (95% Confidence Interval) from univariate regression models.

^2^Values are regression coefficients (95% Confidence Interval) from multivariate regression models containing age, gender, tooth type, arch position, whether caries involves the mesial surface, anesthesia method and restoration technique.

3. Supplementary outcomes: To investigate the impact of IPC in the LA and GA groups, we conducted subgroup analyses stratified by anesthesia method (Table 4). The failure rate in LA group was slightly higher than the failure rate in GA group. In LA group, the failure rate in the 1st primary molar was slightly higher than the 2nd primary molar (HR: 0.31, 95% CI: 0.13–0.79). Meanwhile, the choice of restoration had an observable effect on the success rate of IPC in GA group (HR: 0.13, 95% CI: 0.03–0.63), and the success rate of stainless steel crown was higher than that of resin composite.

**Table 4:** Supplementary subgroup analysis results stratified by anesthesia method (N = 666)

Variables	LA group			GA group		
	Failure	Success	Adjusted HR^1^ (95% CI)	Failure	Success	Adjusted HR^1^(95% CI)
Age (Years), mean (SD)	4.92 (1.19)	5.11 (1.23)		4.17(0.75)	4.33 (1.12)	
Gender, n(%)						
Male	15(3.02)	228(45.87)		4 (2.36)	103(60.95)	
Female	10 (2.01)	244(49.09)		2 (1.18)	60 (35.50)	
Tooth type, n(%)						
1^st^ primary molar	19(3.82)	230(46.28)	Ref.	4 (2.37)	85(50.30)	Ref.
2^nd^primary molar	6(1.21)	242(48.69)	0.31 (0.13–0.79)	2 (1.18)	78(46.15)	0.24(0.03–2.23)
Arch position, n(%)						
Maxillary arch	12(2.41)	243(48.89)		5(2.96)	90(53.25)	
Mandibular arch	13 (2.62)	229(46.08)		1 (0.59)	73(43.20)	
whether caries involves the mesial surface, n(%)						
No	1 (0.20)	64 (12.88)		1 (0.59)	11(6.51)	
Yes	24 (4.83)	408(82.09)		5 (2.96)	152(89.94)	
Restoration, n(%)						
Resin composite	22 (4.43)	365(73.44)	Ref.	2 (1.18)	12(7.10)	Ref.
Stainless steel crown	3 (0.60)	107(21.53)	0.67 (0.37–1.23)	4 (2.37)	151(89.35)	0.13 (0.03–0.63)
Total, n(%)	25 (5.03)	472(94.97)		6(3.55)	163(96.45)	

*LA* Local anesthesia, *GA* general anesthesia, *HR* hazard ratio, *CI* confidence interval, *SD *standard deviation, *Ref* reference

^1^Values are regression coefficients (95% Confidence Interval) from multivariate regression models containing age, gender, tooth type, arch position, whether caries involves the mesial surface, anesthesia method and restoration technique.

## Discussion

IPC is a well-established conservative treatment for deep caries in primary molars, offering the advantages of preserving pulp vitality, minimal invasiveness, and single-visit completion [5, 6, 16]. Despite its recognized benefits, factors influencing its long-term success—particularly the role of anesthesia modality—remain underexplored. This was a retrospective study to examine and compare long-term IPC efficacy under GA and LA in primary molars and evaluate factors affecting success rates. Patients were not randomly assigned to either the GA or LA group, resulting in selection bias. For example, children with more complex conditions, greater dental involvement, or poorer compliance are more likely to be assigned to GA. To minimize bias from non-random grouping, PSM was used in the data analysis to control for confounding factors [17]. We constructed a propensity score-weighted logistic regression model based on confounding variables and used the nearest neighbor matching technique for 1:1 matching. As a result, a total of 120 matched samples were selected from 1,427 teeth of 483 children. As shown in Table 1, after PSM, the characteristics of the two patient groups were balanced. After PSM, all standardized mean differences (SMD) were < 0.20, and *P* > 0.05 for all included covariates. No notable standardized differences were observed for any baseline covariates post-matching. 

Our survival analysis revealed no observable difference in IPC success rates between GA and LA groups. The IPC success rate remained high throughout the observation period, declining gradually from 100.00% at treatment initiation to 93.00% at 40 months. Importantly, the overall survival rates showed no notable time-dependent decrease, regardless of the anesthesia method employed.

These findings indicate that, in appropriately selected cases, the choice of anesthesia does not influence the long-term success of IPC. Consequently, clinical attention may be better directed toward modifiable prognostic factors—such as the use of stainless steel crowns for first primary molars—rather than prioritizing optimized operative settings provided by GA alone.

This study observed IPC success rates exceeding 90.00% with no observable decline over time. This finding aligns with the 100% success rate reported by Kothari et al. [18] in a 12-month trial, although it contrasts with the findings of Dai et al. [14], who observed a decline to 82.3% within 48 months. Similarly, Stratigaki et al. [5] reported 100% clinical and 86.7% radiographic success using calcium hydroxide, further supporting the viability of IPC for deep caries. The favorable long-term outcomes observed in this study may be attributed to rigorous case selection, standardized procedures, and the biological properties of calcium hydroxide. As the core material used, calcium hydroxide creates a high-alkaline microenvironment that eliminates residual bacteria and degrades the extracellular matrix, thereby releasing endogenous bioactive molecules (e.g., transforming growth factor-beta 1 (TGF-β1), bone morphogenetic proteins (BMPs)) that recruit pulp progenitor cells and promote odontoblast-like differentiation [19]. This mechanism is widely considered fundamental to the success of IPC. In addition, calcium hydroxide-treated dentin matrix may exert antibacterial and immunomodulatory effects that further support pulp healing [20, 21]. 

Clinically, the efficacy of calcium hydroxide is well-documented. Long-term studies have reported high success rates for direct pulp capping with calcium hydroxide, exceeding 95% over ten years [22]. Furthermore, comparative studies have shown no observable difference in success rates between calcium hydroxide and calcium silicate-based materials in either direct or indirect pulp capping [23, 24]. Although materials like IRoot BP may promote more pronounced calcific bridge formation, clinical and radiographic outcomes remain comparable across materials, with success rates ranging from 79.4% to 100% [24]. Given these comparable outcomes, calcium hydroxide remains a cost-effective and widely accepted option. The dependence of IPC’s molecular repair process on a sterile environment explains why restoration technique emerged as an independent prognostic factor in our study [25, 26]. Bacterial microleakage can disrupt the signaling pathways essential for pulp stem cell proliferation and mineralization, thereby negating the biological benefits of pulp capping [27–30]. A high-quality, well-sealed restoration is therefore critical, particularly as materials like calcium hydroxide may exhibit physical instability over time [22]. Full-coverage restorations, such as stainless steel crowns, offer superior marginal sealing [30, 31] and are the preferred standard for restoring multisurface cavities in primary molars [32, 33]. By providing this seal, they protect the underlying biological repair process, which explains our observation that stainless steel crowns were associated with higher IPC success rates than composite resins. This finding aligns with the substantial evidence supporting full-coverage restorations in vital pulp therapy [34, 35].

In the multivariate analysis, first primary molars were associated with lower long-term IPC success compared to second primary molars (HR: 0.34, 95% CI: 0.15–0.77). This finding aligns with Maqbool et al. [8], who attributed better outcomes in second primary molars to their larger pulp chamber volume and later eruption. However, Yu et al. [36] reported no such association, a discrepancy that may reflect differences in cohort composition—their study included more GA cases, where optimized operative conditions could potentially attenuate anatomical disadvantages. In our study sample, LA constitutes a substantial proportion, suggesting that the observed tooth-type effect may be more evident in settings with greater procedural variability.

In this study, age, gender, arch position, number of decayed surfaces, and anesthesia method were not observably associated with IPC success. The biological success of IPC is primarily dependent on the pulp’s intrinsic reparative capacity [37, 38]. Within the age range and clinical conditions of this cohort, patient age alone did not emerge as a determinant of outcome, provided the pulp was deemed healthy at treatment initiation. While age-related behavioral factors could theoretically influence treatment success through effects on compliance, such potential confounders were not systematically assessed in this retrospective design. 

The lack of association between anesthesia method and IPC success is consistent with Songvejkasem et al. [39], who reported comparable pulpectomy outcomes under GA and LA. Although some authors have suggested that GA may facilitate more cautious case selection and mitigate technical challenges related to patient cooperation [40], our findings suggest that—with careful case selection and standardized operative protocols—the anesthesia modality itself may have limited independent impact on long-term IPC success.

The strengths of this study include, first, the use of PSM, which minimizes selection bias between the GA and LA groups, thereby enhancing the reliability of conclusions. Second, the study provides meaningful long-term efficacy data based on patients receiving IPC with extended follow-up periods. We acknowledge that IPC under GA represents a specific clinical scenario. While some practitioners may prefer pulpotomy under GA due to complete caries removal feasibility, recent evidence supports IPC as equally effective for deep caries without pulp exposure [14]. Our findings apply specifically to IPC and should not be extrapolated to pulpotomy outcomes.

However, this study has several limitations inherent to its retrospective observational design. First, despite using PSM to balance observed covariates, we cannot fully eliminate indication bias related to general anesthesia assignment. The decision to use GA is inherently based on unmeasured clinical judgments, including patient cooperation, disease severity, parental preferences, and behavioral profiles (e.g., Frankl Behavior Rating Scale, which was not systematically recorded), that are not routinely documented in retrospective archives. Additionally, other unmeasured confounders may systematically differ between groups, such as baseline caries severity, oral hygiene status, treatment compliance, parental oral health literacy, and socioeconomic status. As PSM can only adjust for measured variables, residual confounding from these unobserved factors may persist. Therefore, our findings should be interpreted as associations rather than causal effects. Finally, we recognize that the construction of Directed Acyclic Graphs (DAGs) would have been the preferred approach for covariate selection to minimize bias and ensure valid causal inference. However, given the complexity of causal pathways in our clinical setting and our team’s limited expertise in DAG methodology, we employed the Change-in-Estimate method as a practical alternative. Future studies with access to specialized epidemiological support should consider DAG-informed variable selection to strengthen causal inference

## Conclusion

This study demonstrated that:

1. IPC achieved high long-term success rates in primary molars with deep caries, confirming its efficacy as a conservative treatment option.
2. IPC survival was not associated with anesthesia modality including LA and GA after adjusting for measured confounders. However, prospective validation is required to establish causality.
3. Tooth type and restorative technique were identified as independent prognostic factors. In contrast, age, gender, arch position, decayed tooth surfaces and anesthesia method showed no observable effect.
4. Therefore, clinical practice should focus on strict case selection, standardized procedures, and prioritizing full-coverage restorations (e.g., stainless steel crowns) to optimize pediatric IPC outcomes. Regular long-term follow-up is recommended for all children undergoing IPC.

## Supplementary Information

Supplementary Material 1.

Supplementary Material 2.

## Data Availability

All the data generated and analyzed in this study are available from the corresponding author on reasonable request.

## Declarations

**Clinical trial number**.

## Abbreviations

AAPD: American Academy of Pediatric Dentistry
CI: Confidence interval
ESE: European Society of Endodontology
GA: General anesthesia
HR: Hazard ratio
IPC: Indirect pulp capping
LA: Local anesthesia
PSM: Propensity score matching
SD: Standard deviation
SMD: Standardized mean differences
STROBE: Strengthening the Reporting of Observational Studies in Epidemiology
