# Supplementary material for: Survival of indirect pulp capping in deeply carious primary molars under local versus general anesthesia: a retrospective cohort study using propensity score matching
Source: BMC Oral Health. 2026 Mar 11;26:693. doi: 10.1186/s12903-026-08019-w (PMC13088596; doi:10.1186/s12903-026-08019-w)
Supplement: Supplementary file 2 — Supplementary Material 2. [file 12903_2026_8019_MOESM2_ESM.pdf]

Online Supplementary Material

Survival of Indirect Pulp Capping in Deeply Carious Primary Molars Under Local versus General Anesthesia: A Retrospective Cohort Study Using Propensity Score Matching

Qiyin Sun[1]

Xinling Liang[1]

Mianxiang Li[1]

Affiliations

[1]Department of Stomatology, Guangzhou Women and Children's Medical Center, Guangzhou Medical University, Guangzhou, China

Correspondence to: Qiyin Sun, dentist-sun@foxmail.com

Contents

Figure S1. Histogram of Propensity Score Distribution Before and After Matching
Table S1. Associations of Covariates with IPC Success Rates (N = 666)
Table S2. E-value for The Effect of Anesthesia Methods on The Success Rate of IPC (N = 666)

Fig. S1. Histogram of Propensity Score Distribution Before and After Matching

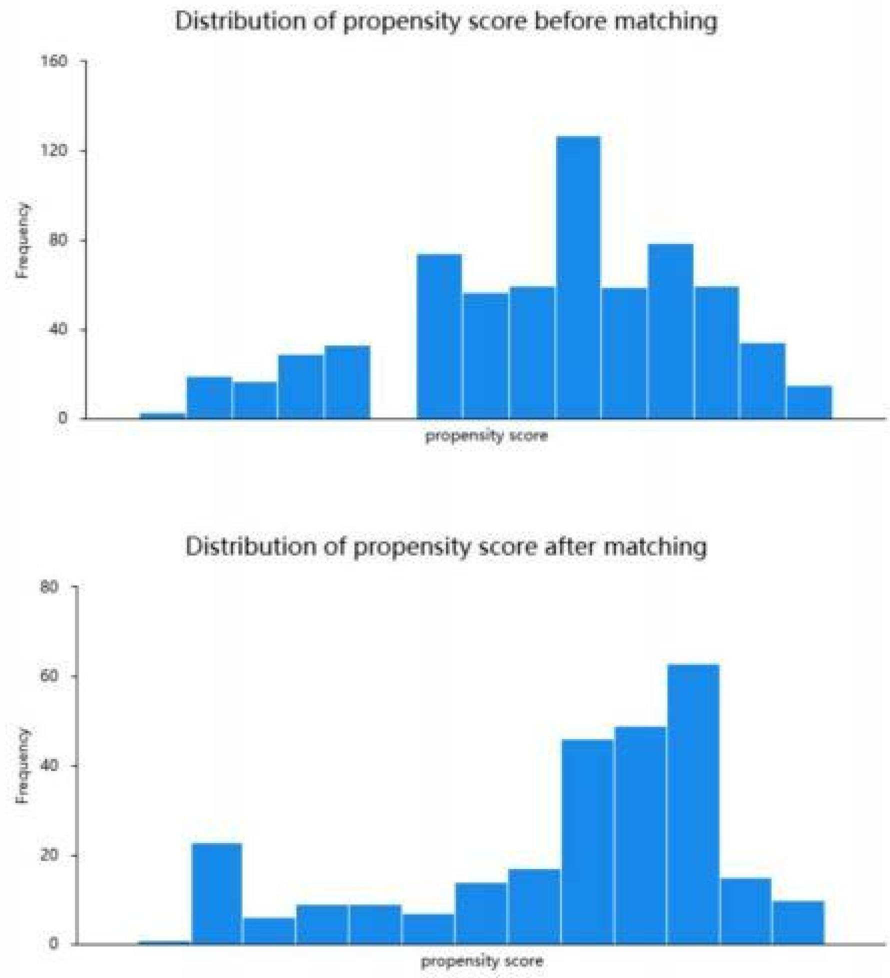

Table S1. Associations of Covariates with IPC Success Rates (N = 666)

	Model1<br>HR(95% CI)	Model2<br>HR (95% CI)	Model3<br>HR (95% CI)
**Age**	.947 (.707 – 1.268)	.911(.671 – 1.236)	.865(.628 – 1.191)
**Gender**			
Male	Ref.	Ref.	Ref.
Female	.707 (.343 – 1.459)	.676(.326 – 1.402)	.620(.296 – 1.296)
**Tooth type**			
1st primary molar	Ref.	Ref.	Ref.
2nd primary molar	.339 (.152 – .758)	.383(.153 – .769)	.343 (.153 – .769)
**Arch position**			
Maxillary arch	Ref.	Ref.	Ref.
Mandibular arch	.921 (.454 – 1.869)	.914(.450 – 1.854)	.792(.385 – 1.629)
**Whether caries involve the mesial surface**			
No	Ref.	Ref.	Ref.
Yes	1.955(.467 – 8.197)	2.055(.489 – 8.641)	2.797(.618 – 12.660)
**Anesthesia method**			
LA	Ref.		Ref.
GA	.695 (.285 – 1.695)		1.723(.386 – 7.681)
**Restoration**			
Resin composite	Ref.	Ref.	Ref.
Stainless steel crown	.656 (.431 – 1.000)	.572(.321 – 1.019)	.478 (.236 – .965)

Abbreviations: IPC, indirect pulp capping; HR, hazard ratio; CI, confidence interval; LA, local anesthesia; GA, general anesthesia

Model1: Univariate regression models.

Model2: Multivariate regression models, containing the independent variable anesthesia method and single covariate.

Model3: Multivariate regression models containing all variables.

Table S2: E − value for the effect of anesthesia methods on the success rate of IPC (N = 666)

Anesthesia method	HR	95% CI	E-value for HR	E − value for CI
LA	Ref.	Ref.	Ref.	Ref.
GA	1.845	.618-5.508	3.09	1.00

Abbreviations: IPC, indirect pulp capping; HR, hazard ratio; CI, confidence interval; LA, local anesthesia; GA, general anesthesia; LL, lower limit of the confidence interval; UL, upper limit of the confidence interval

Calculation formula:

HR >1

HR E-value = HR + √[HR × (HR - 1)]

CI If LL of CI ≤1, then E-value = 1

If LL of CI >1, then E-value = LL +√[LL × (LL - 1)]

HR <1

HR E-value = (1 / HR + √[(1 / HR) × (1 / HR) - 1)]

CI If UL of CI ≥1, then E-value = 1

If LL of CI <1, then E-value = (1 / UL +√[(1 / UL) × (1 / UL) - 1)]
